# Longitudinal Changes of Ocular Surface Microbiome in Patients Undergoing Hemopoietic Stem Cell Transplant (HSCT)

**DOI:** 10.3390/jcm13010208

**Published:** 2023-12-29

**Authors:** Suzanne Clougher, Marco Severgnini, Antonella Marangoni, Clarissa Consolandi, Tania Camboni, Sara Morselli, Mario Arpinati, Francesca Bonifazi, Michele Dicataldo, Tiziana Lazzarotto, Luigi Fontana, Piera Versura

**Affiliations:** 1 Ophthalmology Unit, DIMEC, Alma Mater Studiorum Università di Bologna, 40138 Bologna, Italy; suzanne.clougher@unibo.it (S.C.); luigi.fontana6@unibo.it (L.F.); 2 Institute of Biomedical Technologies—National Research Council, 20054 Segrate, Italy; marco.severgnini@itb.cnr.it (M.S.); clarissa.consolandi@itb.cnr.it (C.C.); tania.camboni@itb.cnr.it (T.C.); 3 Microbiology Unit, DIMEC, Alma Mater Studiorum Università di Bologna, 40138 Bologna, Italy; antonella.marangoni@unibo.it (A.M.); sara.morselli6@unibo.it (S.M.); tiziana.lazzarotto@unibo.it (T.L.); 4 IRCCS Azienda Ospedaliero—Universitaria di Bologna, 40138 Bologna, Italy; mario.arpinati@unibo.it (M.A.); francesca.bonifazi@unibo.it (F.B.); michele.dicataldo@studio.unibo.it (M.D.)

**Keywords:** ocular surface, microbiome, HSCT, oGVHD

## Abstract

Purpose: To evaluate changes in the ocular surface microbiome (OSM) between pre- and post-haemopoietic stem cell transplant (HSCT) in the same patient, and to assess the potential impact of these changes in ocular graft-versus-host disease (o)GVHD development. Methods: Lower fornix conjunctival swabs of 24 patients were obtained before and after HSCT and subjected to DNA extraction for amplification and sequencing of the V3-V4 regions of the bacterial 16S rRNA gene. The obtained reads were reconstructed, filtered, and clustered into zero-radius operational taxonomic units (zOTUs) at 97% identity level before taxonomic assignment, and biodiversity indexes were calculated. Transplant characteristics were recorded, and dry eye was diagnosed and staged 1–4 according to the Dry Eye WorkShop (DEWS) score. Results: No significant difference in OSM alpha diversity between pre- and post-transplant was found. A significant difference in beta diversity was observed between patients with a DEWS score of 1 versus 3 (*p* = 0.035). Increased corneal damage between pre- and post-HSCT was significantly associated with a decrease in alpha diversity. The changes in OSM were not associated with oGVHD, nor with any transplant parameter. Conclusions: This preliminary study is the first study to analyse changes in the OSM before and after HSCT longitudinally. No trend in OSM biodiversity, microbial profile, or overall composition changes before and after HSCT was significant or associated with oGVHD onset. The great variability in the observed OSM profiles seems to suggest the absence of a patient-specific OSM “signature”.

## 1. Introduction

Graft-versus-host disease (GVHD) is the major complication of allogeneic (allo-) hematopoietic stem cell transplantation (HSCT), which severely impacts the survival [1] and quality of life [2] of patients. The gastrointestinal tract is particularly affected by GVHD, and the onset of dysbiosis of the intestinal microbiota has been demonstrated in these patients, characterized by a diversity loss, a decrease in commensals, and an increase in pathogens [3,4]. Recent scientific evidence suggests that the composition of the gut microbiota may be a predictive biomarker for the risk and severity of acute GVHD and the survival of patients undergoing allo-HSCT [5,6], and that gut microbial alterations persist during late complications [7]. However, the mechanisms underlying these associations are not yet well understood, and the same can be said of the clinical strategies to be applied to modulate the microbiome and reduce the transplant complications.

Ocular manifestations of GVHD (oGVHD) are frequent post-transplant complications that develop in 30–60% of patients after allo-HSCT and 60–90% of patients with acute or chronic systemic GVHD [8,9,10]. It can affect all the structures of the eye, resulting in reduced visual acuity [11] and restricted daily activities [12]. Dry eye disease (DED) is the hallmark of ocular chronic GVHD (oGVHD) and is used for its diagnosis, scoring, and prognosis assessment [13,14,15]. There is no evidence so far of a link between the gut microbiome and ocular surface microbiome, although a gut–eye axis has been hypothesized and the role of gut microbiota on ocular disease onset has been extensively analyzed [16].

Despite its exposition to the external environment, the ocular surface hosts a paucibacterial microbiome [17,18] mainly composed of three phyla [19,20,21], recently renamed Pseudomonadota, Actinomycetota, and Bacillota, and appears to play a role in regulating immune responses [22]. At the genus level, combined data from 11 studies on healthy adult volunteers point toward a core ocular surface microbiome (OSM) including *Corynebacterium*, *Acinetobacter*, *Pseudomonas*, *Staphylococcus*, *Propionibacterium*, and *Streptococcus* [19]. Several studies showed shifts in the OSM of patients affected by different pathologies, such as dry eye disease, diabetes mellitus, and allergic conjunctivitis [23,24,25].

The characterisation of OSM in oGVHD patients has been published in a few papers, with different clinical designs and applying different analytical techniques. Shimizu et al. found by conventional culture techniques that among 40 post-HSCT patients, GVHD patients showed more diversified microbial communities in their conjunctiva compared with the nonGVHD patients and the controls [26]. Two other studies characterised the OSM of post-allogenic HSCT patients with or without oGVHD through metagenomic shotgun sequencing and reached opposite results, as a decrease in the OSM diversity of post-HSCT patients compared to healthy controls was reported in both [27,28]. This could be due to the heterogeneous populations included in the studies and the use of different techniques, as the first study used traditional culture methods and the other two opted for next-generation sequencing methods.

As our group suggested in previous papers, the evaluation of ocular surface parameters in patients either before or after HSCT is essential to drive clinical decisions regarding oGVHD patient management [10,29]. The aim of this study was to compare the pre- and post-transplant OSM of patients undergoing HSCT, in order to evaluate the possible longitudinal changes at an individual level, as well as the relations between OSM composition and diversity, and the development or not of oGVHD.

## 2. Materials & Methods

### 2.1. Subjects

This single-centre study included data from 24 subjects (6 females and 18 males) affected by haematological malignancies, who had undergone a pre-HSCT and a post-HSCT complete clinical ocular examination as briefly described below, in the period between December 2019 and June 2021. Clinical data were retrieved from charts in the frame of a non-interventional observational study authorized by the local Ethical Committee and performed in accordance with the Declaration of Helsinki. Exclusion criteria were no ocular surgery and no use of topical antibiotic or steroids in the six months prior to HSCT; allergy; and use of contact lenses.

### 2.2. Haematological Characteristics

Factors considered were recipient sex and age at transplantation, diagnosis (acute lymphoblastic leukemia, acute myeloid leukemia, or myelodysplastic syndrome), source of stem cells (bone marrow versus peripheral blood stem cells), donor age at transplantation, type of donor (matched related or unrelated), donor–recipient sex matching, human leukocyte antigen (HLA) compatibility (HLA-A and HLA-B and high-resolution typing for HLA-DRB1), type of conditioning (myeloablative or reduced-intensity regimen based on patient age, previous treatments, comorbidities, and status of malignancy), and anti-T Lymphocyte globulin (ATLG) use. All the patients underwent GVHD prophylaxis using both systemic cyclosporine and methotrexate or cyclosporine and mycophenolate mofetil.

### 2.3. Ocular Examination and Sampling

Schirmer I test and tear film break-up time (TFBUT) were performed according to the DEWS guidelines [30]; evaluation of subjective symptoms was scored with the ocular surface disease index (OSDI) questionnaire [31] and by visual analogue scale (VAS); evaluation of corneal damage by fluorescein dye and conjunctival damage by lissamine green dye staining were scored with the National Eye Institute (NEI) score [32]; Ocular Protection Index (OPI) [33] was performed with the CA-800 Topographer (Topcon Healthcare Italy, Milan, Italy); meibomian gland loss was evaluated automatically in percentage on a CA-800 Topographer in the lower lid; and conjunctival scraping cytology was evaluated [34] with a score ranging by severity 0–20 (cut off value > 5). DED was staged according to the method proposed by DEWS [35] by increasing levels of severity from 1 to 4, whereas a score of 0 meant no DED.

Each patient underwent two examinations and sampling of the OSM, one pre-HSCT and one post-HSCT. The pre-HSCT examination was performed before the conditioning regimen and the post-HSCT examination within 6 to 12 months postoperatively, according to the management established for these patients in our Unit. All the patients had received a therapy in the first month post-operation, based on 0.2% hyaluronic acid or 3.0% trehalose-based tear substitutes, which was prolonged in fourteen out of twenty-four patients to relieve transient subjective discomfort.

The diagnosis of ocular GVHD was based on the International Consensus Criteria on Chronic Ocular GVHD Group, which assigns a scoring point of 0–3 to the Schirmer test, corneal fluorescein staining, and OSDI, and a scoring point of 0–2 to conjunctival injection [36].

### 2.4. Sampling, DNA Extraction, 16S rRNA Sequencing, and Processing

Lower fornix conjunctival swab specimens were collected (E-swab, Copan, Murrieta, CA, USA) in both eyes of all the patients, under one drop of topical anaesthesia with 0.4% oxybuprocain. The collected samples were immediately transported and stored in a −20 °C freezer until DNA extraction.

Before DNA extraction, the two swabs from the same subject were pooled together. The DNA was isolated from each sample using a DNeasy Blood & Tissue Kit (Qiagen, Hilden, Germany) following the “Pre-treatment for Gram-Positive Bacteria” protocol and the “Purification of Total DNA from Animal Tissues Spin-Column Protocol”. The extracted DNA was eluted twice in 100 µL of Buffer AE (10 mM Tris-Cl, 0.5 mM EDTA; pH 9.0) and stored at −20 °C until being sent for sequencing.

The V3-V4 hypervariable regions of the bacterial 16S rRNA gene were amplified according to the 16S metagenomic sequencing library preparation protocol (Illumina, San Diego, CA, USA). In a total volume of 25 μL, 12.5 ng genomic DNA, 5 µL of forward and reverse primers, and 12.5 μL of 2× KAPA HiFi HotStart ReadyMix were used for each sample. The PCR conditions were as follows: initial denaturation at 95 °C for 3 min, then 25 cycles of 95 °C for 30 s, 55 °C for 30 s, and 72 °C for 30 s; final extension: 72 °C for 5 min. A “blank” control (i.e., a PCR performed with the same Illumina-suggested primers but no DNA template) was added to exclude environmental and lab-related contaminants as the source of the bacteria observed in samples characterised by a low abundance of bacteria, such as in the ocular environment. Final indexed libraries were prepared by equimolar (4 nmol/L) pooling, denaturation, and dilution to 6 pmol/L before loading onto the MiSeq flow cell (Illumina). A 2 × 300 bp paired-end run was performed.

Raw sequencing reads were, first, processed and filtered through a pipeline comprising the merging of the two overlapping forward and reverse reads for each pair by PandaSeq [37] and the trimming/filtering of low-quality bases/reads by the split_libraries_fastq.py script in QIIME v.1.9.0 [38]: reads with stretches of at least 3 bases with Phred quality score < 3 were trimmed from the 3′-end; fragments with a length <75% of the initial fragment length were discarded. Zero-radius operational taxonomic units (zOTUs) were created denoising the filtered reads by USEARCH v. 11.0.667 [39] and retaining only those supported by 5 or more reads. Taxonomic assignment of zOTUs was performed by RDP classifier [40] against the SILVA 138 database [41] using a 0.8 confidence threshold.

### 2.5. Statistical Analysis

Downstream statistical analyses (including alpha and beta diversity evaluations) were performed in QIIME 1.9.0 suite and Matlab (v. 2008a, Natick, MA, USA). Alpha diversity analysis was performed using several metrics (i.e., Shannon’s diversity, Chao1 diversity index, observed species, and Faith’s phylogenetic diversity index). A non-parametric permutation-based *t*-test with 999 random permutations was employed to determine differences between experimental classes. Beta diversity analysis was based on unweighted and weighted UniFrac distances [42] and represented by principal coordinate analysis (PCoA). A permutational multivariate analysis of variance using distance matrices using pseudo-F ratios (“adonis” test of R package “vegan”, https://cran.r-project.org/package=vegan accessed on 20 December 2023) was used to define whether there was a significant difference among the experimental groups, using 999 random permutations. Analysis of the microbial composition of the samples was performed by uploading the data to the Galaxy web platform, and the public server at galaxy.biobakery.org (accessed on 28 September 2023) was used to perform the linear discriminant analysis effect size (LEfSe) [43] and setting DEWS scores as classes (i.e., the main condition under investigation) and pre- and post-transplant time points as subclasses, in order to account for eventual differences due to the diverse microbial communities before and after transplant.

Spearman correlations between hematological characteristics and ocular surface parameters, and alpha diversity indexes were calculated; a *p*-value < 0.05 was considered significant.

## 3. Results

Baseline hematological characteristics are shown in Table 1. The median time between the pre- and post-transplant ocular examination was 164 ± 72 days (ranging between 70 and 303 days). Eight out of twenty-four patients were diagnosed with oGVHD.

Pre- and post-HSCT differences in ocular parameters are summarized in Table 2. Subjective symptoms of discomfort scored by the VAS scale, scraping cytology index scoring inflammation, corneal damage, and the ocular protection index significantly worsened in the post-HSCT examination as compared to the pre-HSCT one. Tear stability and Meibomian gland loss were already found to be in the pathological range pre-HSCT; both were reduced (although not significantly) in the post-HSCT evaluation.

In the pre-HSCT evaluation, only one patient did not show any sign of DED (DEWS score = 0); the remaining patients were diagnosed as suffering from DED at various severity levels and scored as DEWS = 1 (n = 7), DEWS = 2 (n = 14), and DEWS = 3 (n = 2). Four patients worsened from a DEWS score of 2 to 3 in the post-HSCT.

A total of 3,013,187 sequencing reads were obtained for the 48 total samples (1 sample pre- and 1 sample post-HSCT for each of the 24 patients), with an average of 62,775 ± 81,214 reads per sample. After fragment rebuilding, quality filtering, and zOTUs creation, the dataset comprised 2,719,392 reads belonging to 4354 zOTUs (average of 56,654 ± 76,955 reads per sample). For all the subsequent analyses, each sample was rarefied to the least sequenced one (n = 4157) to have an even representation of the microbial composition of the whole dataset. This number of reads was sufficient to describe the main features of the ecosystem, as demonstrated by analysing the rarefaction curves of the alpha diversity metrics for an increasing number of reads per sample (Supplementary Figure S1), and to continue the analysis.

From a taxonomic point of view, the most abundant phyla in the dataset were *Bacillota*, *Pseudomonadota*, *Actinomycetota*, and *Bacteroidota*, with substantial differences from sample to sample. For example, the relative abundance of Bacillota varied between 1.2% and 99.8% and the one of Actinomycetota between <0.1% and 41.4%, but this behaviour was evident also for all the other phyla; on average, 2.2% of the relative abundance was due to bacteria unclassified at lower taxonomic levels. Notably, sample 7B was composed almost exclusively of Pseudomonadota (Supplementary Figure S2A). The same profile was reflected at a family level, with *Staphylococcaceae*, *Moraxellaceae*, *Corynebacteriaceae*, *Pseudomonadaceae*, and *Sphingomonadaceae* as the main constituents (average relative abundance >2% over the whole dataset); sample 7B showed a profile nearly completely composed of *Pseudomonadaceae* (Supplementary Figure S2B).

**Figure 1:**
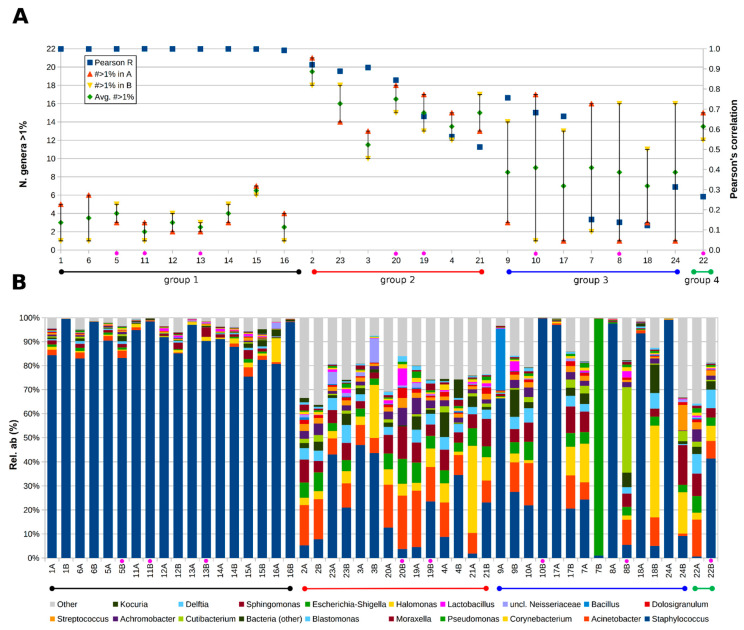
(**A**) Plot of pre- and post-HSCT samples correlation and variability. For each patient, the number of genera having an abundance >1% in pre- (sample (**A**) or post-HSCT (sample (**B**) is represented, together with the average between the two. On the secondary Y-axis, the Pearson’s correlation score between the microbial composition of pre- and post-HSCT samples from the same patients based on the 19 most relatively abundant genera is shown. Less abundant taxa are grouped in the “Other” category. (**B**) Bacterial composition at genus level for the samples is paired per each patient. Only the 19 most abundant genera are shown, whereas less abundant taxa are grouped in the “Other” category. Samples from individuals affected by ocular GVHD are indicated by a purple circle in both panels (**A**,**B**).

Interesting features were pointed out considering together the microbial profiles of the samples at genus level and the correlation between pre- (point A) and post-HSCT (point B) samples from the same patients. In fact, pairing Pearson’s correlation coefficients with the number of genera having an abundance >1% in pre- and post-HSCT samples, we highlighted four groups of patients (Figure 1).

The first group was composed of patients whose A and B points were highly correlated (correlation coefficient close to 1) and with a low-variable microbiota, in which the number of genera with an abundance >1% was less than 6 and was dominated by *Staphylococcus* (patients: 1, 6, 5, 11, 12, 13, 14, 15, and 16). A second group composed of patients whose A and B points were moderately or strongly correlated (correlation coefficient between 0.5 and 0.9) and with a more variable microbiota, in which the number of genera with an abundance >1% was between 10 and 20 (patients 2, 23, 3, 20, 19, 4, and 21). The third group of patients consisted of those whose A and B points greatly varied in terms of microbial composition (patients 9, 10, 17, 7, 8, 18, and 24). Here the pre- and post-HSCT microbiota of the same patient did not resemble each other, generally with one dominated by *Staphylococcus* and the other more varied. Among them, the high correlation observed for some patients (i.e., 9, 10, and 17), characterized by a very different pre- and post-HSCT microbiota, was due to an artifact linked to a single taxon (i.e., *Staphylococcus*) that increased the correlation, which otherwise would have been very low. In this group, patient 7 showed a pre-HSCT microbiota with a diversified composition, whereas the post-HSCT one (sample 7B) was entirely composed of *Pseudomonas*. Finally, one last patient (i.e., patient 22) was characterised by a varied microbiota in both time points (number of genera with abundance >1% more than 12), but with a low correlation coefficient (r = 0.27), due to the different compositions of the two paired samples.

Regarding the microbial compositions of the samples at genus level, the two main profiles were either dominated by *Staphylococcus* (24 samples, average relative abundance: 90.2%), with a negligible contribution of other taxa (all having an average abundance ≤1%), or had a more diversified microbiota (23 samples, excluding sample 7B), made up of *Staphylococcus* (19.0%), *Acinetobacter* (12.2%), *Corynebacterium* (9.4%), *Pseudomonas* (5.4%), *Moraxella* (7.3%), *Blastomonas* (4.2%), *Cutibacterium* (3.0%), and other genera adding up to 39.6% of the total relative abundance on average. The blank control was composed of very few reads (i.e., 20), suggesting that no outside contamination was detected in the sequenced samples.

**Figure 2:**
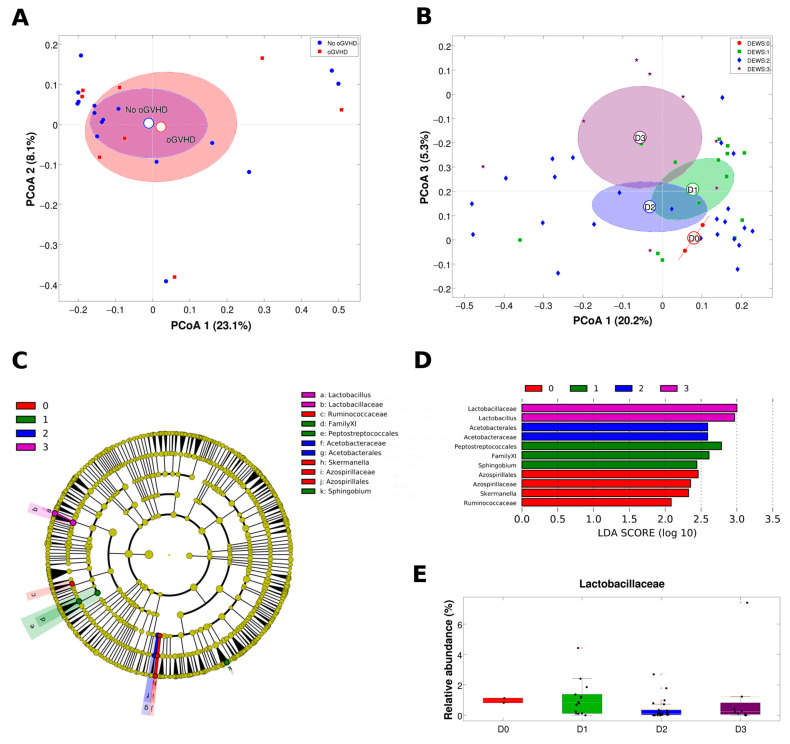
(**A**,**B**) Principal coordinates analysis (PCoA) based on the unweighted UniFrac distances among samples with data points coloured according to diagnosis of oGVHD (**A**) or DEWS score (**B**). Each point represents a sample; centroids (reported as outlined circles in the middle of ellipses) are the average of sample coordinates within the same experimental category; ellipses are the SEM-based confidence intervals. Coordinates 1 and 3 (**A**) or 1 and 2 (**B**) are represented. (**C**) Cladogram of taxa and (**D**) bar plots of the LDA scores for the taxa significantly associated with DEWS score from the linear discriminant analysis effect size (LEfSe) analysis of microbial composition. Colours indicate the DEWS score associated with each taxon. (**E**) Boxplot of the relative abundance of members of the *Lactobacillaceae* family in the samples grouped according to DEWS score; median is represented in black and mean in orange; values for individual samples are reported as black circles.

The microbial profiles of the post-HSCT samples were analysed comparing samples from patients who developed oGVHD to those from patients who did not. We found no significant difference in either biodiversity (alpha diversity, Supplementary Figure S3B, Supplementary Table S1) or composition (beta diversity, Figure 2A) of the two experimental classes for all the metrics tested. However, interesting results were highlighted classifying the samples (both pre- and post-HSCT samples) according to the DEWS score. Samples with a DEWS score =1 showed a trend (although not statistically significant) to be more biodiverse than samples with DEWS = 2 or DEWS = 3 (Supplementary Figure S3A). At the same time, the microbial profiles were statistically different (unweighted UniFrac metric, Adonis test, *p* = 0.048) according to the DEWS score; in particular, pairwise comparisons between categories revealed that DEWS = 1 samples were significantly different from those with DEWS = 3 (unweighted UniFrac metric, Adonis test, *p* = 0.035, Figure 2B). Although the centroids in the PCoA plots seemed well separated, no difference was recorded for the DEWS = 2 vs. DEWS = 3 comparison, probably due to the dispersion of DEWS = 2 data points with a very heterogeneous microbial composition. The category DEWS = 0 was not evaluable, since it only contained 2 samples.

Considering the taxonomic composition of the samples (Figure 2C,D), DEWS = 3 was characterised by a lower content of *Lactobacillaceae* (Figure 2E) and *Lactobacillus* (median abundance: 0.2%, compared to 1.0% and 0.6% in DEWS = 0 and DEWS = 1, respectively); DEWS = 2 samples showed a low content of *Acetobacteriaceae* (median abundance: 0.1%), whereas DEWS = 1 samples had a higher abundance of Family XI of *Peptostreptococcales* (median abundance: 0.3%, compared to <0.05% of other categories) and *Sphingobium*. Finally, DEWS = 0 samples showed a presence of *Skermanella* and Ruminococcaceae (median abundances: 0.05% and 0.12%, respectively, whereas these genera were not found in samples with DEWS = 1 to 3).

**Figure 3:**
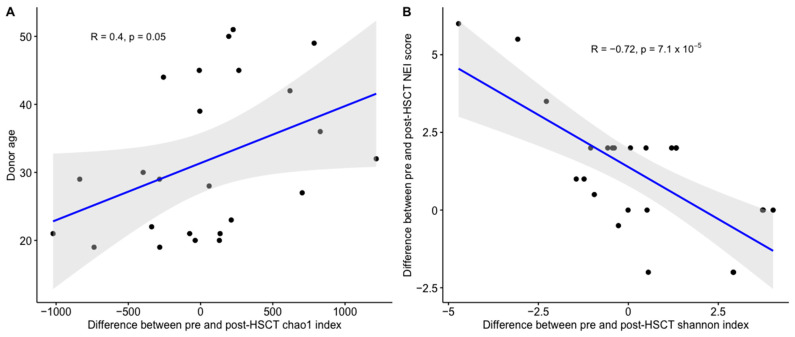
(**A**,**B**) Correlation indexes between alpha diversity and age/NEI.

No statistical significance was found in the correlation analysis between hematological, ocular parameters and microbiome diversity indices, except for the following two. A direct correlation between donor age and alpha diversity (Chao1 and Shannon index) was found (Figure 3A). An inverse correlation between pre- and post-HSCT increases in NEI corneal damage and alpha diversity (Shannon index) was shown (Figure 3B).

## 4. Discussion

This preliminary study is the first to analyse longitudinal changes in the OSM before and after HSCT in the same patient and showed no significant change in the OSM biodiversity trend, microbial profile, or overall composition, irrespective of the development or not of oGVHD. The microbial profiles were different according to the DEWS severity score, with a lower content of *Lactobacillaceae* in the more severe DED patients.

The current interest in the role of the microbiome focuses on its potential relation with clinical outcome in patients undergoing hematologic transplantation. This aspect was specifically evaluated with regard to the gut microbiome, and still little is known on the influence eventually exerted by the OSM on oGVHD development. A few previous studies compared healthy subjects with post-HSCT patients who had or had not developed oGVHD, reporting inconsistent results in terms of OSM diversity indexes and composition [26,27,28]. The discrepancies in these studies’ results are possibly due to cross-sectional trial design matching heterogeneous populations and differences in methodology. As mentioned, the present study longitudinally evaluates OSM in the same patients, a more scientific approach to assessing changes related to transplantation, as suggested and previously shown in other ocular surface parameters by our group [10,29,44,45,46].

Data from the present study shows that OSM diversity indexes and relative abundances do not significantly change from pre- to post-HSCT as a whole. In our cohort, the main genera identified in post-HSCT patients are *Staphylococcus*, *Corynbeacterium*, *Pseudomonas*, *Chryseobacterium*, in agreement with those reported in the two previous studies on the same issue [27,28]. An attempt to better document and group different OSM compositions according to the correlation between pre- and post-HSCT highlighted four different profiles, but no significant correlation was found.

In general, high microbial diversity is regarded as a positive indicator of health [47] suggesting how low diversity may induce greater fragility of the system. However, this issue is still under debate, as increased diversity might also be associated with dysbiosis and disease onset, data that are possibly derived from studies of small-scale cohorts of patients [48]. For OSM, the importance of microbial diversity is still not fully understood. Our data did not show any diversity index decrease related to the presence of oGVHD, a condition strongly impacting the ocular surface system, but found a correlation to increased corneal damage post-HSCT. This finding appears in agreement with a previous study [49] showing that an altered OSM is present in the eyes of patients with more severe corneal damage (i.e., traumatic ulcers), but further data are needed to determine whether this might impact the disease progression. Data from the present study also showed an increase in OSM alpha diversity index correlated with increased donor age; this finding appears to be at odds with the fact that better clinical outcomes are reached with younger donors, although the parameters to identify the ideal donor are still in progress [50].

Microorganisms detected in the present study appear to be in the trend of what is reported in the previous literature on OSM. The most abundant phyla in our patient cohort were *Bacillota*, *Pseudomonadota*, and *Bacteroidota* although with different profiles compared to the previous papers on post-HSCT patients [26,27,28]. The same consideration can be drawn at a family level, with *Staphylococcaceae*, *Moraxellaceae*, *Corynebacteriaceae*, *Pseudomonadaceae*, and *Sphingomonadaceae* as the main constituents. As an element for discussion, without it being the purpose of this paper, the role of OSM in general has not yet been well characterised even in normal subjects, although several papers have been published so far. These studies, however, are hard to compare because of the many differences in sampling procedures, sample preparation and analysis, type of analytical instruments and settings used, and statistical analysis.

Ocular surface is susceptible to cytotoxicity from numerous chemotherapeutic agents and targeted therapies used before HSCT [51], and previous studies have demonstrated that dry eye disease is already present in a significant percentage of patients before transplantation, as we have also previously shown [29,52]. In the present cohort, all but one patient had dry eye disease (DED) before transplantation and, in agreement with previous studies, a pre-post HSCT worsening in several ocular parameters has been shown [53,54]. Data from this study shows that OSM profiles in patients suffering from severe DED (DEWS = 3) post-HSCT showed prevalent families comparable with those found in less severe patients (DEWS = 1 and DEWS = 2), with a notable exception for the abundance of *Lactobacilli*. This observation might be intriguing, since *Lactobacillaceae* were found to be mostly expressed in healthy eyes [55], and *Lactobacillus*-based probiotic mixtures have been successfully proved to improve Sjögren’s syndrome-associated dry eye [56].

The weakness of the present study consists primarily in the number of enrolled patients, although the design of the pre-post HSCT longitudinal analysis strengthens the consistency of results and brings data to the still limited investigation on the issue. A gender bias may also have been introduced, as the study was composed of 18 males and 6 females and a difference between male and female OSM alpha diversity [27] and beta diversity [57] has been reported in previous studies. The post-HSCT evaluation was performed in a period of six to twelve months after transplant, which has neglected the effects of the antibiotic therapy which the HSCT patients were subjected to; on the other hand, the peak of changes is said to be in the period between three to six months, so perhaps an evaluation performed in a period closer to the surgery would bring different results.

As a conclusive comment, data from this study would suggest that the OSM is well controlled in patients closely monitored and already managed before HSCT, and even almost a year post-surgery there has been no change with overriding harmful or damaging species.

## Figures and Tables

**Table 1:** Baseline hematological characteristics of the study cohort.

Characteristics	Value
Recipient sex (age)	
Females n.	6 (57.5, 18–65)
Males n.	18 (52, 24–68)
Diagnosis	
ALL	4 (16.7%)
AML	12 (50%)
APLASTIC ANEMIA	1 (4.2%)
MDS/MPN	5 (20.8%)
NHL	2 (8.3%)
Source of stem cells	
Bone marrow	3 (12.5%)
Peripheral blood stem cells	21 (87.5%)
Donor age	28.5 (19–51)
Type of donor	
Matched unrelated donor	17 (70.8%)
Matched related donor	7 (29.2%)
Donor–recipient sex matching	
Female to male	11 (45.8%)
Others	13 (54.2%)
HLA compatibility	
Matched	12 (50%)
Mismatched (≥1 antigen)	12 (50%)
Type of conditioning	
Myeloablative	15 (62.5%)
Reduced intensity	9 (37.5%)
ATLG	21 (87.5%)

ALL: acute lymphoblastic leukemia; AML: acute myeloid leukemia; MDS/MPN: Myelodysplastic syndrome/myeloproliferative neoplasm; NHL: non-Hodgkin’s lymphoma. HLA: Human leukocyte antigen. ATLG: anti-T Lymphocyte globulin. Age is expressed in years, median and min–max value.

**Table 2:** Results from ophthalmological examinations in patients before and after HSCT.

Parameters	Pre-HSCT	Post-HSCT	*p*
OSDI score	11.1 ± 15.7	14.5 ± 14.3	0.33
VAS	1.4 ± 2.2	2.4 ± 2.3	0.03
TFBUT (sec)	7.2 ± 2.6	6.6 ± 3.4	0.40
Schirmer test (mm length/5′)	13.8 ± 7.7	17.9 ± 11.2	0.08
Conjunctival staining (van Bijsterveldt score)	2.1 ± 2.4	2.8 ± 2.9	0.31
Corneal damage (NEI score)	1.2 ± 0.8	2.3 ± 1.9	0.01
MGD score (%)	36.3 ± 10.2	33.5 ± 7.3	0.32
Scraping cytology score	3.6 ± 1.8	5.5 ± 2.8	0.01
OPI	1.4 ± 1.0	1.0 ± 0.6	0.01

OSDI = ocular surface disease index; VAS = visual analogue scale; TFBUT = tear film break-up time; NEI = National Eye Institute. MGD = meibomian gland dysfunction; OPI = ocular protection index. Values are expressed as mean ± SD. Paired *t*-test *p*-value.

## Data Availability

Raw sequencing data are available from the NCBI Short Read Archive (SRA) under BioProject PRJNA1048377 (https://www.ncbi.nlm.nih.gov/bioproject/PRJNA1048377, accessed on 20 December 2023).

## Supplementary Materials

The following supporting information can be downloaded at: https://www.mdpi.com/article/10.3390/jcm13010208/s1, Figure S1: Alpha diversity rarefaction plots according to sample ID; Figure S2: Ocular microbiota composition at phylum (A) and family (B) level; Figure S3: Alpha diversity rarefaction plots according to DEWS score (A) and presence/absence of oGVHD (B); Table S1: Differences pre-HSCT VS post-HSCT of alpha diversity indexes.
