# Supplementary material for: Longitudinal Changes of Ocular Surface Microbiome in Patients Undergoing Hemopoietic Stem Cell Transplant (HSCT)
Source: J Clin Med. 2023 Dec 29;13(1):208. doi: 10.3390/jcm13010208 (PMC10779677; doi:10.3390/jcm13010208)
Supplement: Supplementary file 1 [file jcm-13-00208-s001.zip › jcm-2662022-supplementary/CLOUGHER_suppl_Table S1.pdf]

Supplementary Table S1: Differences pre VS post of alpha diversity indexes.

Mean, Median (min; max) [95% CI], p.value (Welch two sample t-test).

oGVHD= ocular Graft Versus Host Disease

Indexes	oGVHD	Not oGVHD	p. value
Shannon index	-0.44<br>-0.42 (-3.07; 1.33)<br>[-3.07; 1.33]	0.39<br>0.28 (-4.72; 4.02)<br>[-1.23; 2.91]	0.3317
Chao1 index	-185.7<br>-179.8 (-836.5; 225.5)<br>[-836.50; 225.53]	80.78<br>27.13 (-1020.88; 1215.82)<br>[-282.68; 619.08]	0.2394
No. of observed species	-118.83<br>-93.00 (-642.00; 179.00)<br>[-642; 179]	60.12<br>-23.5 (-728.00; 848.00)<br>[-171; 540]	0.2774
PD whole tree	-2.07<br>-0.89 (-8.81; 1.80)<br>[-8.81; 1.80]	0.51<br>0.09 (-7.10; 14.49)<br>[-4.51; 3.47]	0.2517
