# Supplementary figures and images for: Longitudinal Changes of Ocular Surface Microbiome in Patients Undergoing Hemopoietic Stem Cell Transplant (HSCT)
Source: J Clin Med. 2023 Dec 29;13(1):208. doi: 10.3390/jcm13010208 (PMC10779677; doi:10.3390/jcm13010208)

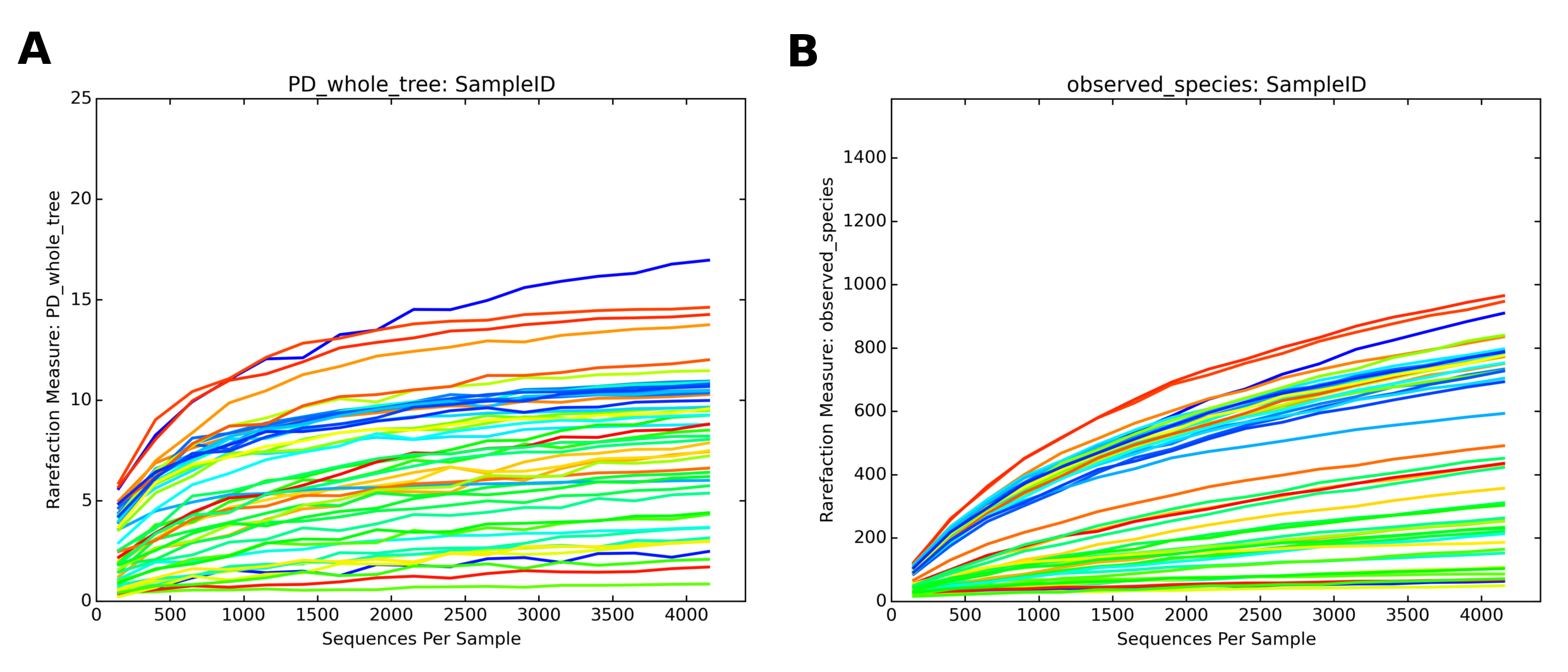

Supplement: Supplementary file 1 [file jcm-13-00208-s001.zip › jcm-2662022-supplementary/CLOUGHER_suppl_Figure S1.tiff]

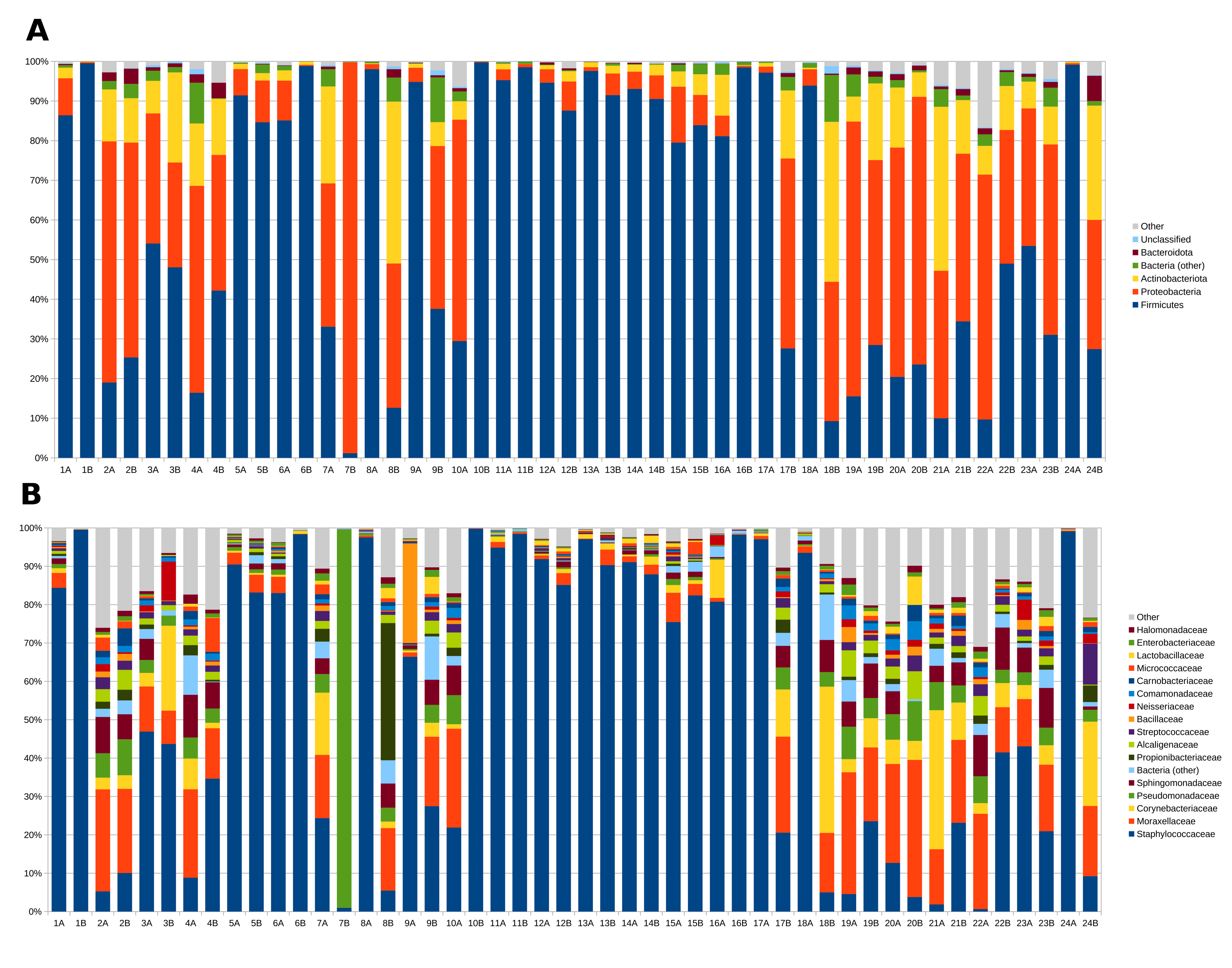

Supplement: Supplementary file 1 [file jcm-13-00208-s001.zip › jcm-2662022-supplementary/CLOUGHER_suppl_Figure S2.tiff]

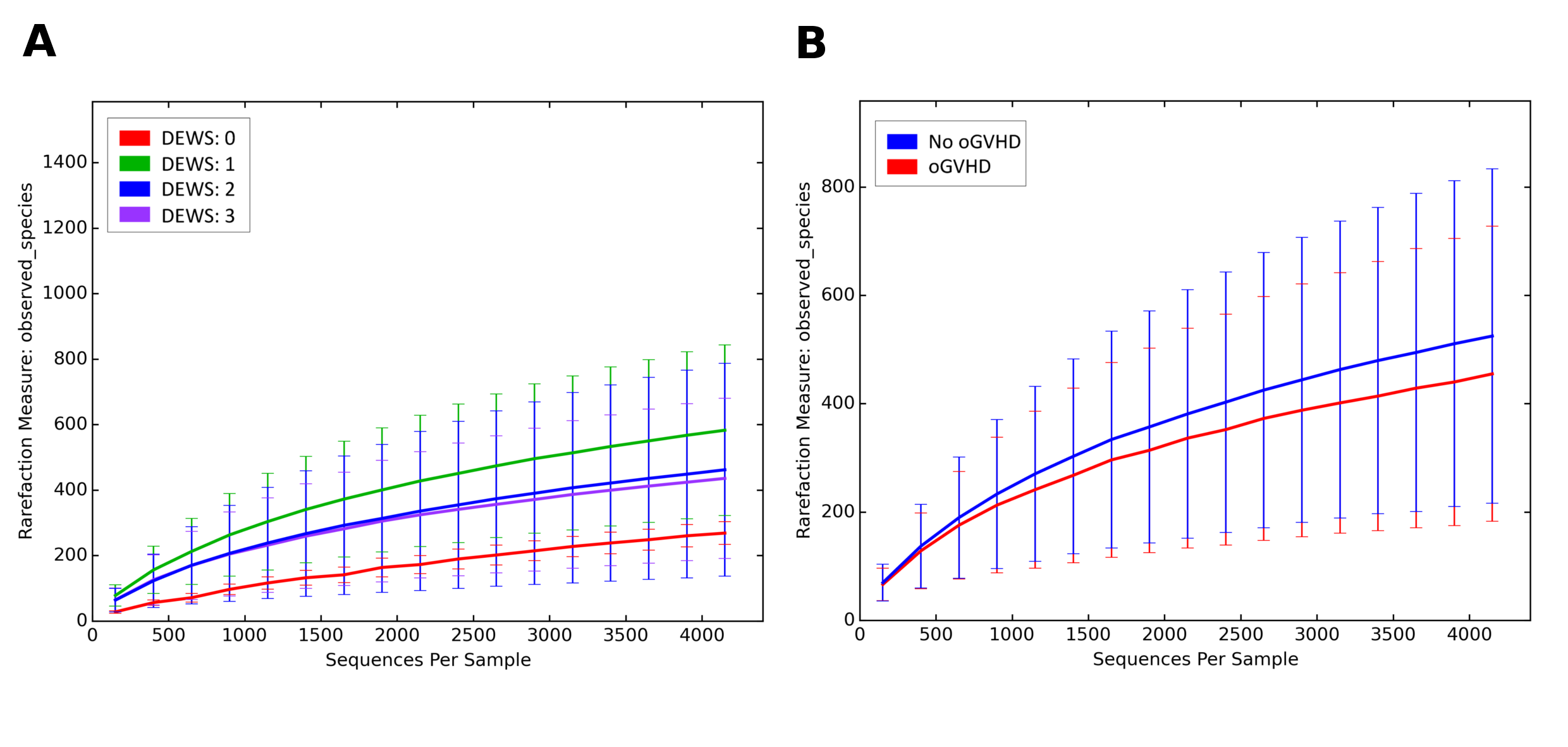

Supplement: Supplementary file 1 [file jcm-13-00208-s001.zip › jcm-2662022-supplementary/CLOUGHER_suppl_Figure S3.tiff]
